# Response to: Brazilian guideline for pediatric cycloplegia and mydriasis

**DOI:** 10.5935/0004-2749.2023-0264

**Published:** 2023-11-24

**Authors:** Goksu Alacamli

**Affiliations:** 1 Ophthalmology Departmant, Trakya University Faculty of Medicine and Education and Research Hospital, Edirne, Turkey

Dear Editor,

We read the article by Ian Curi, Simone Akiko Nakayama, Érika Mota Pereira, Luisa Moreira Hopker, Fábio Ejzenbaum, Ronaldo Boaventura Barcellos, Rosane da Cruz Ferreira, Monica Fialho Cronemberger, Craig A. Mckeown, and Júlia Dutra Rossetto^(1)^ with interest. Responding to this comprehensive, well-thought-out article^(1)^, I would like to highlight a serious side effect of cycloplegic and mydriatic drops used in retinopathy of prematurity (ROP) examinations for premature infants. Though instances of babies experiencing this side effect are rare and have only been reported in past case studies, necrotizing enterocolitis (NEC) should be considered in infants showing gastrointestinal symptoms such as abdominal distension, pneumatosis intestinalis, and ongoing abdominal color change after the application of mydriatic eye drops (cyclopentolate, tropicamide, and phenylephrine). 20%–40% of NEC patients require surgery, with a significant mortality rate^(2,3)^. Also, isolated case reports have previously presented transient ileus associated with the use of mydriatics after screening for retinopathy of prematurity in low birth weight infants^(4,5)^. Although these cases^(2,3,4,5)^ are rare, abnormal abdominal and gastrointestinal findings should prompt pediatricians or ophthalmologists to diagnose NEK or ileus. Practitioners dealing with these immature infants must also be aware of these potential complications.

At our clinic, Trakya University Faculty of Medicine and Education and Research Hospital, we administer 0.5% tropicamide and 2.5% phenylephrine 30 minutes prior to the ROP examination. We have the opportunity to purchase these drops in low doses commercially. In our tertiary referral faculty of medicine hospital, we experience low rates of side effects from the ROP examination due to these types of drops. The article^(1)^ describes that cyclopentolate is generally not preferred due to its high side effect rates in infants.

The article by Curi et al.^(1)^ provides a comprehensive and detailed guideline for pediatric cycloplegia and mydriasis. We owe immense gratitude and respect to all authors^(1)^.

Although, phenylephrine and tropicamide collyrium are well tolerated in neonates, to minimize side effects, the concentrations of these eye drops should be limited (cylopentolate at 0.5%, phenylephrine at 2.5%, tropicamide at 0.5%). The drug excess should be cleaned up. Application of pressure to the eye’s medial corner will avoid nasal mucosal absorption of the topical eye drop^(1,2,3,4,5)^.

Author Contributions: Significant contribution to conception and design: Goksu Alacamli. Data Acquisition: Goksu Alacamli. Data Analysis and Interpretation: Goksu Alacamli. Manuscript Drafting: Goksu Alacamli. Significant intellectual content revision of the manuscript: Goksu Alacamli. Have given final approval of the submitted manuscript (mandatory participation for all authors): Goksu Alacamli. Statistical analysis: No statistical analysis. Obtaining funding: None. Supervision of administrative, technical, or material support: Goksu Alacamli. Research group leadership: Goksu Alacamli.
