# Supplementary material for: Response to: Brazilian guideline for pediatric cycloplegia and mydriasis
Source: Arq Bras Oftalmol. 2023 Nov 24;87(1):e2023-0264. doi: 10.5935/0004-2749.2023-0264 (PMC11575741; doi:10.5935/0004-2749.2023-0264)
Supplement: Related texts

# Reply: Response

**Authors:** Julia D. Rossetto

Dear Dr. Alacamli,

We appreciate your interest in our article, your shared knowledge of your service’s practices, and your careful attention to the severe side effects of phenylephrine. All ophthalmologists should know the side effects of a commonly used ophthalmic drug that can be highly detrimental to premature infants.

In the work, we emphasize these effects in the section “Should we use phenylephrine as an adjuvant to maximize mydriasis?” as follows:

Special attention should be paid to extremely premature infants, very low-weight infants, and patients with respiratory distress, as they are more prone to gastrointestinal side effects. In these patients, blood supply vasoconstriction and anticholinergic effects can decrease peristalsis, leading to delayed gastric emptying, emesis, abdominal distension, and even necrotizing enterocolitis. In older infants and children, using one drop of 2.5% phenylephrine, though seldom required, offers a satisfactory safety profile. However, a 10% concentration appears to cause a significant rise in the number and severity of side effects, including reports of cardiorespiratory arrestt^(1)^.”

The comment aptly highlights the signs to watch for such side effects: abdominal distension, pneumatosis intestinalis, and abdominal color change.

Additionally, we would like to thank the valuable references mentioned in your comment.

Julia D. Rossetto

Corresponding author

## References

[1] Curi I, Nakayama SA, Pereira ÉM, Hopker LM, Ejzenbaum F, Barcellos RB (2023). Brazilian guideline for pediatric cycloplegia and
mydriasis. Arq Bras Oftalmol [Internet].
